# Cystitis: From Urothelial Cell Biology to Clinical Applications

**DOI:** 10.1155/2014/473536

**Published:** 2014-04-30

**Authors:** Gilho Lee, Rok Romih, Daša Zupančič

**Affiliations:** ^1^Department of Urology, Dankook University College of Medicine, 359 Manghyang-ro, Cheonan 330-715, Republic of Korea; ^2^Institute of Cell Biology, Faculty of Medicine, University of Ljubljana, Vrazov trg 2, SI-1000 Ljubljana, Slovenia

## Abstract

Cystitis is a urinary bladder disease with many causes and symptoms. The severity of cystitis ranges from mild lower abdominal discomfort to life-threatening haemorrhagic cystitis. The course of disease is often chronic or recurrent. Although cystitis represents huge economical and medical burden throughout the world and in many cases treatments are ineffective, the mechanisms of its origin and development as well as measures for effective treatment are still poorly understood. However, many studies have demonstrated that urothelial dysfunction plays a crucial role. In the present review we first discuss fundamental issues of urothelial cell biology, which is the core for comprehension of cystitis. Then we focus on many forms of cystitis, its current treatments, and advances in its research. Additionally we review haemorrhagic cystitis with one of the leading causative agents being chemotherapeutic drug cyclophosphamide and summarise its management strategies. At the end we describe an excellent and widely used animal model of cyclophosphamide induced cystitis, which gives researches the opportunity to get a better insight into the mechanisms involved and possibility to develop new therapy approaches.

## 1. Introduction

Cystitis is inflammation of the urinary bladder with diverse and often unknown etiology. Our understanding of cystitis rests in the comprehension of currently insufficient but rapidly growing knowledge about structure-function relationships within urinary bladder and its interaction with other organ systems, especially with nervous system. Urinary bladder wall is composed of three layers: (i) the mucosa, (ii) the muscularis propria, and (iii) the adventitia/serosa. The mucosa contains the urothelium, the epithelium which faces the urine, basal lamina, which separates urothelium from underlying connective tissue, and lamina propria. Lamina propria is composed of an extracellular matrix containing several types of cells, including fibroblasts, myofibroblasts/interstitial cells, immune cells, and afferent and efferent neurons. In addition, lamina propria contains blood and lymphatic vessels, elastic fibres, and smooth muscle fascicles (muscularis mucosae). Muscularis mucosa is not very well defined in the human bladder and sometimes seems to be absent [1]. Muscularis propria is formed by the detrusor muscle, which is organised into three layers of smooth muscle fibres running in different directions. Urothelium lines not only the inner surface of the urinary bladder, but also the renal pelvis, ureters, and proximal urethra [2]. The urothelium of the urinary bladder is composed of three distinctive cell layers. Functionally, it forms a high-resistance permeability barrier (blood-urine barrier) to molecules and ions as well as pathogens in the urine and can accommodate to large changes in urine volume during micturition cycles [3]. Terminally differentiated superficial cells, called umbrella cells, are responsible for maintaining the blood-urine barrier, which depends on two structures: tight junctions with the highest resistance in the mammalian body [4] and the apical plasma membrane with unique specializations named urothelial plaques [5]. Furthermore, these cells are resistant to large mechanically deforming forces such as stretch (during filling and storage) and sudden compression (during voiding), which is accomplished by their high foldability and capacity to alter their apical surface area by exocytosis and endocytosis [6]. Moreover, the urothelium acts as an integral part of the urinary bladder sensory web, which receives, amplifies, and transmits information to the underlying tissues including sensory nerve fibres, myofibroblasts, and smooth muscle cells [7]. In this respect, urothelium releases various mediators and neurotransmitters to reflect its degree of physical distension, so that both sympathetic and parasympathetic nerves can coordinate normal bladder function during filling and voiding [8]. The permeability barrier and sensory function of the urothelium are compromised in various diseases that affect the urinary bladder. For example, it is proposed that in patients with neurogenic detrusor overactivity lower permeability barrier of the urothelium might lead to enhanced signalling responsible for urinary frequency and bladder pain [9]. Similar events are observed in cystitis, where lower permeability barrier of the urothelium could be directly correlated with defective differentiation of urothelial cells [4].

## 2. Urothelial Differentiation and Formation of Blood-Urine Permeability Barrier

**Figure 1:**
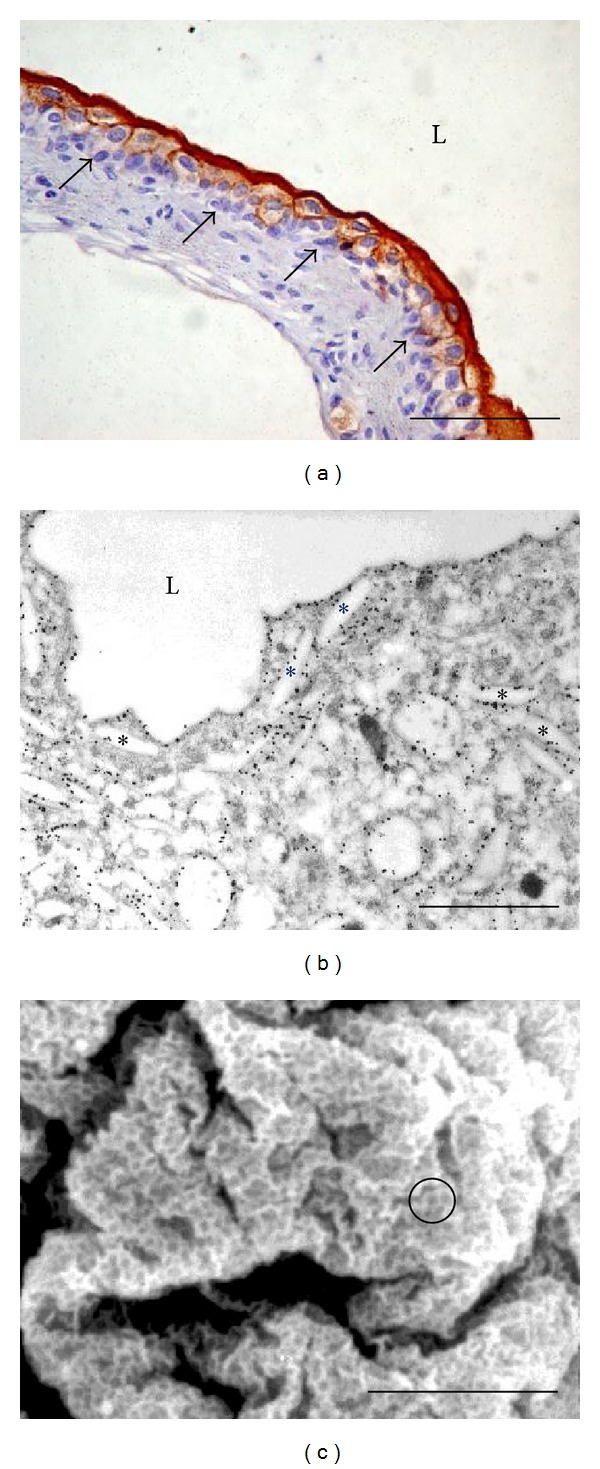
Normal mouse urinary bladder with terminally differentiated umbrella cells in the superficial layer of the urothelium. (a) Immunolabelling on paraffin section shows strong expression of uroplakins in umbrella cells (dark brown), weaker expression in intermediate cells (light brown), and negative basal cells that lie on the basal lamina (arrows). (b) Immunolabelling on ultrathin section demonstrates uroplakins (12 nm colloidal gold particles, black) in the membranes of fusiform vesicles (asterisks) and in the apical plasma membrane facing bladder lumen (L). (c) Scanning electron microscopy of the umbrella cell shows that its apical plasma membrane is covered with urothelial plaques (grey) interconnected by hinges (white). One such plaque and hinge region is encircled. Scale bars (a) = 50 *μ*m, (b) = 1 *μ*m, and (c) = 10 *μ*m.

The function of the urothelium as an effective blood-urine barrier is accomplished by its normal differentiation process. Differentiation runs from basal cell layer, facing the basal lamina, across intermediate to the superficial cell layer, which is in contact with urine. Basal cells are small and they can divide mitotically. Some of the basal cells are urothelial stem cells but their identification remains controversial because of the lack of specific markers [10]. It has been proposed that 9% of basal cells represent putative urothelial stem cells in rat urothelium [11]. Above basal cell layer towards the lumen of the urinary bladder there are intermediate cells. The thickness of intermediate cell layer differs between mammalian species; in rodents it is one cell layer thick, while in human it is up to six cell layers thick. Intermediate cells in rodents start to express urothelium-specific proteins, uroplakins (UPs; Figure 1(a)) [12, 13]. However, UPs are detected primarily in the superficial umbrella cells in human urothelium [14]. UPs belong to a group of evolutionary conserved integral membrane proteins that comprises four major members, UPIa (27 kDa), UPIb (28 kDa), UPII (15 kDa), and UPIIIa (47 kDa) [15, 16]. UPIa and UPIb belong to the tetraspanin family, while UPII and UPIIIa have a single transmembrane domain. All four UPs have large extracellular domains, which gives the urothelial membranes a thickened (12 nm) asymmetric appearance, readily seen with transmission electron microscope [17]. UPs appear in dimers, namely, UPIa/UPII and UPIb/UPIIIa heterodimers. These heterodimers associate to form heterotetramers and such six heterotetramers are assembled into a 16 nm intramembrane particle [18]. Hexagonally packed 16 nm particles form two-dimensional crystals known as urothelial plaques, which are interconnected by thinner membranes known as hinges [19]. In intermediate cells of rodents, UPs are present in the membranes of cytoplasmic vesicles, called fusiform vesicles, but not in the plasma membrane [20]. In superficial umbrella cells, terminal urothelial differentiation is achieved. Umbrella cells have high levels of UPs expression (Figure 1(a)), which is reflected in the formation of large urothelial plaques in post-Golgi compartments [21]. Two urothelial plaques form each fusiform vesicle, which is therefore flattened in shape (Figure 1(b)). Usually 4–15 fusiform vesicles are joined together into stacks [20]. Such shape and organisation of fusiform vesicles make them a perfect storage compartment, which can transport large amounts of urothelial plaques to the apical plasma membrane of umbrella cells. It is believed that fusiform vesicles fuse with the apical plasma membrane during distension of the urinary bladder. This exocytotic event is not completely understood in umbrella cells but it was proposed that cytokeratins, Rab27b, and MAL protein play important roles [22, 23]. Urothelial plaques cover 70–90% of the urothelial apical surface, which can be demonstrated by scanning electron microscopy (Figure 1(c)), and they represent structural basis for blood-urine barrier.

The expression of UPs and the presence of urothelial plaques are therefore two main characteristics for establishing urothelial differentiation and also for predicting functional, high-resistance permeability barrier [24, 25]. Moreover, UPs are also suggested as useful markers for diagnosis, detection, and prognostic prediction of urothelial carcinomas [26].

## 3. Cystitis and Advances in Its Research and Patients Care

Cystitis can be clinically described as a syndrome of dysuria, urgency, frequency, and lower abdominal pain. Although cystitis is usually caused by bacterial infection, it can also be caused by noninfectious conditions such as carcinoma in situ, bladder cancer, and bladder stone or it can even emerge from unknown origin as in interstitial cystitis [27]. Urologists usually distinguish cystitis of infectious origins and of noninfectious origins. The category of infectious cystitis can further be classified into uncomplicated cystitis and complicated cystitis (Table 1). Uncomplicated cystitis can be described as an infection in women with a structurally and functionally normal urinary bladder. However, complicated cystitis is associated with structurally or functionally abnormal urinary bladder where the host is compromised and pathogens develop antimicrobial resistance. After careful differential diagnosis, appropriate treatment must be used, which results in successful management in most cystitis instances [28, 29].

Most cases of cystitis occur in women. In addition, each year approximately 10% of all women report a urinary tract infection and more than 50% of all women have at least one such urinary bladder infection in their lifetime [27, 30]. The symptoms of cystitis are very variable but usually painful urination, urgency, frequency, lower abdominal pain, and haematuria can develop (Table 1). Presence of clinical symptoms or signs is sufficient to diagnose uncomplicated cystitis in addition to simple urine analysis with microscopic findings and gram staining. Urine culture in every patient with the infection is usually recommended [27, 31]. Some of the patients may experience recurrent cystitis [32]. The definition of recurrent cystitis is two or more symptomatic cystitis episodes over a 6-month period or three or more cystitis episodes within a one-year period (Table 1) [27, 33]. Reinfection and bacterial persistence are two typical phenotypes in recurrent cystitis. In healthy women recurrent cystitis is usually caused by reinfection with new pathogens or different pathogens from outside the urinary tract and is classified as the category of uncomplicated cystitis [34]. Nevertheless, recurrent cystitis in compromised men is caused by the same pathogens from the same site within the urinary bladder due to bacterial persistence. This kind of recurrent cystitis is classified as chronic infection and usually occurs in structurally or functionally abnormal urinary bladder and can therefore be classified into the category of complicated cystitis [27, 34]. It is commonly caused by various pathogens with antimicrobial resistance [27] and the patients with chronic cystitis usually have various additional complicating factors, which contribute to the infection [29]. Uncomplicated cystitis usually occurs at variable intervals by different species, while chronic infection is due to the same organism at very close time intervals.

The most common pathogen in uncomplicated and complicated cystitis is uropathogenic* E. coli *(UPEC) strain, followed by* Staphylococcus saprophyticus*, enterococci, coagulase-negative staphylococci, and other species of Enterobacteriaceae [27, 35]. The pathogenesis of UPEC in host cells has been relatively well documented [35, 36]. UPEC strains originate from host's large intestine. However, in contrast to intestinal* E. coli* strains, UPEC strains have a number of virulence factors that enable them to invade into urothelium and survive against host defences [35, 37]. UPEC strains from intestine can adhere to and colonize the perineum and vagina and subsequently migrate to the urinary tract where they cause an inflammatory response in the urothelium [38, 39]. In addition, the increased epithelial receptivity for* E. coli* on the genitourinary organs can be associated with recurrent cystitis [40]. Almost all of UPEC strains express type 1 fimbriae and its adhesin, FimH, enables them to attach to urothelial surface receptor and invade into urothelium of the urinary bladder [37, 41]. Furthermore, UPEC strains typically express an array of toxins such as siderophores for iron acquisition systems and hemolysin and cytotoxic necrotizing factors for exploiting host nutrients and facilitating bacterial dissemination [35, 37]. UPEC strains gain a foothold in the urinary tract by binding FimH to uroplakin UPIa [36, 38]. Seeking intracellular refuge within urothelial cells is the only way that UPEC can avoid elimination by the voiding of urine from the urinary bladder or by the host's innate immunity [36, 38, 42]. Upon ligation of the UPEC to UPIa, widespread conformational changes within the apical plasma membrane of umbrella cells are induced, followed by engulfment of the UPEC into sanctuary [6, 38]. This bacterial invasion is mediated by localized host actin rearrangement and phagocytosis of the bound UPEC by zippering of the membrane around the microorganism [6, 43, 44].

Blocking the binding between UPIa of umbrella cells and FimH of bacteria is an ideal target for infectious cystitis treatment. Specific targeting of the FimH adhesion could be achieved by using the soluble receptor analogues or mannosides that act as antiadhesives. These molecules bind FimH and prevent it from interacting with host receptors [45]. Moreover, it has been reported that surfactant protein D (SP-D) inhibits bacterium-induced cytotoxicity by preventing adherence of UPEC to the umbrella cells and dampen UPEC-induced inflammation in mice [46]. However, we must point out a concern regarding the systemic administration of either mannosides or pilicides that are potentially adversely affecting commensal* E. coli* and other members of the intestinal microbiota, many of which also express type 1 pili [47].

Invasion into the umbrella cells allows UPEC to establish a new niche in an effort to protect itself from the host innate immune response [39, 43]. The intracellular UPEC can multiply within umbrella cells intracellular compartment to form the so-called intracellular bacterial communities, some of which can then switch into a quiescent phase to persist in the cells indefinitely. Intracellular quiescent nature of these bacteria provides their resistance to antibiotics and protects them from host neutrophils and other host surveillance systems [39, 43, 48, 49]. Intracellular signals, such as the reorganization of the actin filaments, can trigger the resurgent growth of UPEC, prompting the development and dispersal of intracellular bacterial communities leading to the recurrence of clinical symptoms [32, 50]. Recently, it has been proposed that the resurrection of these quiescent forms of UPEC is coincident with recurrent cystitis or bacterial persistence [51]. The urothelial cells must therefore prevent the UPEC attacks to survive. Upon contact with UPEC or their products, the host immune surveillance molecules evoke a variety of immune responses aimed at the early elimination of the invading uropathogens. There are many evidences that toll-like receptors (TLRs) are the major contributing factors to the immunogenic resistance of the urinary tract to this microbial attack. Mutant mice with inactive TLR4 are defective in their ability to clear urinary tract infections [52, 53]. This defect is attributed to the inability of urothelial cells to evoke an appropriate cytokine response to uropathogens, which results in limited recruitment of neutrophils to sites of infection in the urinary tract [53].

Even though the natural course of uncomplicated cystitis is usually self-limited and is spontaneously healed, the oral antibiotic agents are the first choice for its treatment [27, 32]. Empirical antibiotics that reveal less than 20% drug resistance among* E. coli* strains are usually recommended. Trimethoprim or trimethoprim and sulfamethoxazole have been widely used as effective and inexpensive agents for empirical therapy in the most areas of the world [27]. In some areas where high resistance to trimethoprim or trimethoprim and sulfamethoxazole has been observed, fluoroquinolone antibiotics are recommended as an alternative drug. Women with recurrent cystitis usually require careful consideration of medical history for the risk factors of reinfection and must consider long-term medical suppressive management [33, 54]. Spermicide use for birth control or for prevention of sexually transmitted infections can be associated with an increased risk of cystitis and vaginal colonization with* E. coli* [55]. Because spermicides with nonoxynol-9 may lead to reduction of vaginal lactobacilli, the preventive mechanisms against bacterial interference can become weak and therefore enhancement of the adherence of* E. coli* strains to vaginal epithelial cells occurs [55, 56]. In addition, the lack of estrogen in menopause women also causes marked changes in the vaginal microflora, including a loss of lactobacilli and increased bacterial colonization [57]. Sexual intercourse in women is also one of the risk factors for cystitis. Women with recurrent cystitis usually require low dose of continuous prophylaxis, self-start intermittent therapy, or postintercourse prophylaxis [27, 54, 55].

Complicated cystitis is the one that occurs in a patient with a compromised urinary tract or that is caused by a very resistant pathogen [29]. These infections are usually caused by an atypical and broad range of bacteria with resistance to multiple antibiotics. Urine cultures, therefore, are mandatory to identify the bacteria and decide for appropriated antimicrobial agents. Patients with chronic cystitis can usually be cured of the recurrent infections by identification and surgical removal or correction of the focus of infection [27]. In addition, functional or structural abnormalities should be corrected, and urinary tract function must be restored by medical, pharmacologic, or surgical management.

## 4. Haemorrhagic Cystitis and Its Treatment

Haemorrhagic cystitis (HC) is defined by urinary bladder irritation signs and haematuria. The disease can be triggered by many circumstances including going through chemotherapy, receiving radiation therapy, and experiencing various bacterial and viral infections [59, 60]. The severity of HC has been reported to range from asymptomatic microscopic haematuria to life-threatening haematuria [61]. The clinical courses of HC are variable depending on the causes. The HC induced by infection is usually self-limited and resolves spontaneously or with appropriate antibiotic therapy. In some patients, however, the removal of the urinary bladder is necessary to save their lives, since life-threatening haematuria from anticancer agents sometimes cannot be controlled by conventional medical methods [62]. Additionally, physicians do not prescribe full therapeutic doses of anticancer agents in the treatment of cancers because severe urologic side effects of these agents have frequently been reported [63].

**Figure 2:**
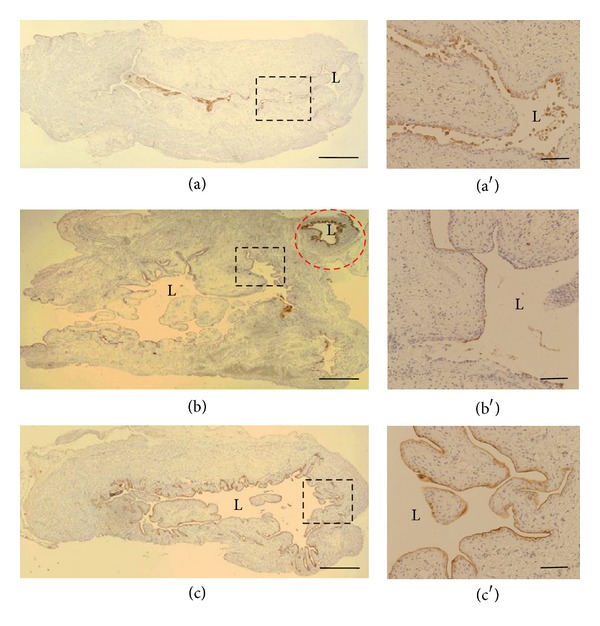
Epinephrine treatment preserves UPII expression in rat urinary bladder 24 hours after cyclophosphamide injection. UPII expression (brown) is a well-established transitional urothelial marker that is strongly expressed along the mucosal area in dilated ureter (red circle). (a) Cyclophosphamide injected rats showed a decrease or loss of UPII expression. (b) Urethral obstructed and null-treated rats at 24 hours after cyclophosphamide injection revealed a significant decrease or loss of UPII expression. (c) Intravesical epinephrine treated rats after cyclophosphamide injection showed much better expression pattern of UPII along the bladder mucosa. L: lumen of the urinary bladder or of the ureter. Scale bars (a–c) = 1000 *μ*m and (a′–c′) = 200 *μ*m. Reprinted from Kyung et al., 2012 [58], with permission of Springer-Verlag.

Cyclophosphamide (2-[bis(2-chloroethyl)amino]tetrahydro-2H-1,2,3-oxazaphosphorine 2-oxide) was first introduced as an antineoplastic agent in 1958 [64] and since then numerous reports have been published concerning haemorrhagic cystitis, a side effect not observed with other alkylating agents. Currently cyclophosphamide is still widely used in chemotherapy of B cell malignant diseases and some solid tumours, conditioning before bone marrow transplantation, and in the treatment of certain immunoinflammatory conditions, for example, Wegener's granulomatosis, rheumatoid arthritis, and systemic lupus erythematosus [65, 66]. Cyclophosphamide side effects depend on the dosage of cyclophosphamide used and can affect up to 75% of the patients receiving a high intravenous dose. The frequent side effects of cyclophosphamide in the urinary bladder range from irritative voiding symptoms, urinary frequency, dysuria, urgency, suprapubic discomfort, and strangury, with microhematuria, to the potentially life-threatening complication of haemorrhagic cystitis [63, 67]. Cyclophosphamide is metabolized in the liver and possibly in the kidney to 4-hydroxy metabolites (e.g., phosphoramide mustard, PAM, and acrolein) which are renally excreted and stored in the urinary bladder until voiding [68, 69]. PAM is the primary chemotherapeutic metabolite but it has minimal effects on the bladder, while acrolein was recognised as the causative agent in cyclophosphamide induced haemorrhagic cystitis [70]. Acrolein is a highly reactive aldehyde and the mechanism by which acrolein reaches the bladder is unclear, although it is suggested that it might be formed in the lumen of the bladder. Effects of acrolein on the bladder wall are contributed to its contact with umbrella cells and include necrosis, desquamation, oedema, ulceration, neovascularization, and haemorrhage [71]. The therapeutic targets in cyclophosphamide induced haemorrhagic cystitis are dysuria or micturition symptoms and massive haematuria. Dysuria, frequent voiding, and urgency may be controlled with medications, but massive haematuria is a life-threatening symptom and should be immediately controlled. Hyperhydration, bladder irrigation, and agents that can detoxify cyclophosphamide such as Mesna (2-mercaptoethane sodium sulphonate) have been the most frequently used prophylactic measures to prevent treatment-related cystitis but are not always effective [72]. In the search for new prevention and treatment approaches hyperbaric oxygen therapy, flavonoids or polyphenols, and melatonin are suggested as supportive treatment, but further studies are required for their translation into clinic [59, 73, 74]. Another promising clinical prophylactic agent is the epinephrine, which is a very important medicine for controlling vascular bleeding and the function of the sympathetic action. Interestingly, epinephrine also decreases the incidence and severity of cyclophosphamide induced cystitis in rats and has even a greater protective effect than Mesna [75]. The research team of Lee has recently reported that intravesical application of epinephrine has an attenuating effect on uroplakin expression, submucosal edema, and hemorrhage in cyclophosphamide induced rat cystitis [58, 76] (Figure 2). Concurrently, intravesical epinephrine preserved both subtypes of alpha1A- and alpha1B-adrenergic receptor expressions in urinary bladder [58]. Before the clinical application of intravesical epinephrine therapy for cyclophosphamide induced haemorrhagic cystitis, one must consider some hypothetical weak points. First, since *α*-adrenergic stimulation produces relaxation in the bladder body and contraction in bladder neck or prostatic urethra [77], delayed voiding or acute urinary retention can occur. Second, to expect optimal therapeutic effects through intravesical instillation therapy, it is very important to hold the intravesically injected epinephrine within the bladder for maximal absorption. However, exposure to prolonged stagnant urine also poses a risk of longer contact with toxic metabolites of cyclophosphamide. Third, vigorous diuresis or continuous urinary bladder irrigation and frequent urination cannot sustain the therapeutic dosage of intravesically instilled epinephrine and can therefore weaken the effect of the treatment [58].

The first experimental study of cyclophosphamide induced bladder toxicity was that of Philips et al. [78]. In this study it was concluded that urotoxicity is due to contact between the urothelium and cyclophosphamide metabolites in the urine. In rats approximately 70% of the metabolites of the drug are excreted in the urine within 4 h after administration of a single intraperitoneal dose [69]. Although single intraperitoneal injection of cyclophosphamide causes reversible urothelial hyperplasia with gradual restoration of normal three-layered urothelium [69, 79, 80], repeated doses can lead to premalignant and ultimately to malignant transformation [81, 82]. Moreover, it is known that patients treated with cyclophosphamide have up to a ninefold increased risk of developing bladder cancer [83, 84].

## 5. Experimental Models of Haemorrhagic Cystitis

**Figure 3:**
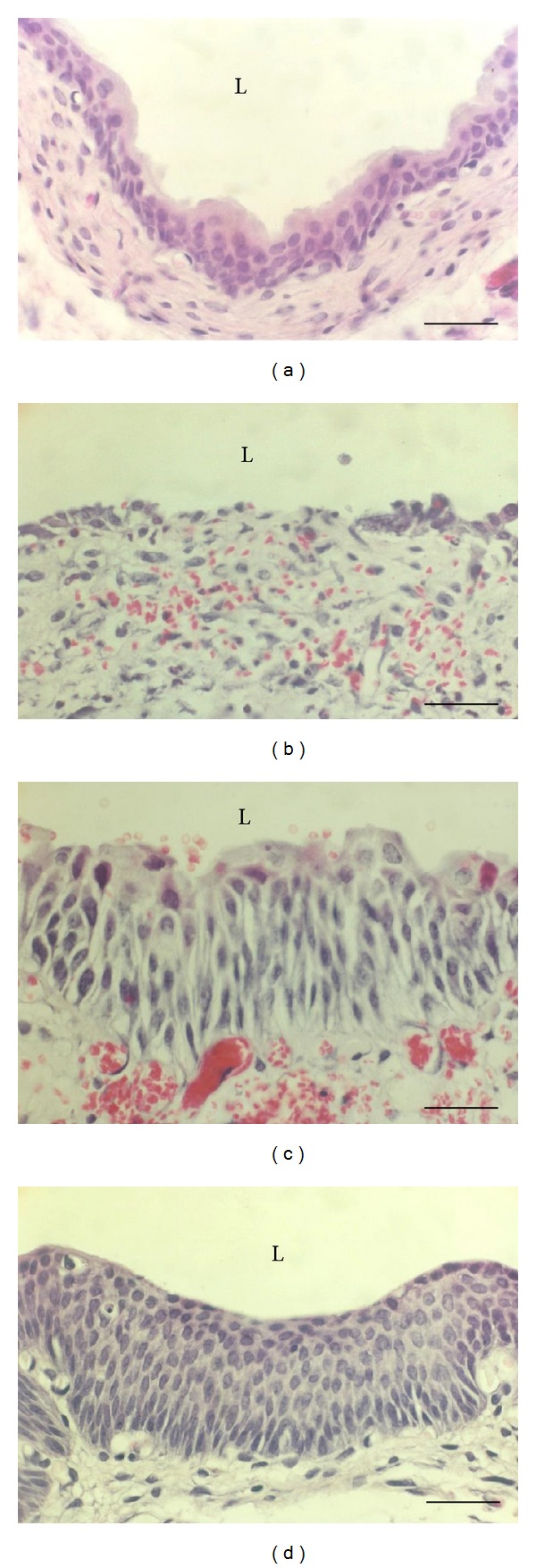
Cyclophosphamide induced changes of the rat urothelium. (a) Normal three-layered urothelium. (b) Urothelium on day 1 after cyclophosphamide injection with some remaining urothelial cells and with denuded areas. Haemorrhage is evident. (c) Urothelium on day 5 after cyclophosphamide injection is hyperplastic, with enlarged intercellular spaces and marked haemorrhage. (d) Urothelium on day 10 after cyclophosphamide injection is hyperplastic and no haemorrhage is seen. L: lumen of the urinary bladder. Scale bars = 50 *μ*m.

An animal model of cyclophosphamide induced haemorrhagic cystitis is one of the best described and methodically developed models. With minor modifications it is currently widely used experimental tool for investigation of pathogenesis, prevention, and treatment of haemorrhagic cystitis as well as urothelial injury, bladder inflammation, bladder-related pain, and acute and chronic overactive bladder [58, 59, 76, 81, 85, 86]. An immediate effect of cyclophosphamide metabolites is seen as widespread destruction of the urothelium, which is accompanied not only by necrosis but also by apoptosis of urothelial cells, with only a few surviving cells remaining after 24 hours (Figure 3) [78, 87]. The surviving cells retain their ability to proliferate and reepithelialize denuded areas [78, 79, 86]. It seems that EGF initiates cell proliferation by binding to EGFR and rapid proliferation of remaining urothelial cells leads to hyperplastic urothelium formation [80]. It should be noted that the normal bladder urothelium is unresponsive to EGF from urine because of the absence of epidermal growth factor receptors (EGFRs) from the superficial layer. Cyclophosphamide exposes partially differentiated urothelial cells that express EGFR in their plasma membranes, which enables urinary EGF to stimulate proliferation. Reversible hyperplasia develops already by days 2 and 3 after cyclophosphamide injection (Figure 3), while gradual restoration of a normal three-layered urothelium is achieved within 2 to 3 weeks [69, 79, 80]. Hyperplastic urothelium enables fast resealing of the injury and represents the key mechanism for the maintenance of functional permeability barrier of the urothelium lacking umbrella cells [88]. The main mechanisms for restoration of a normal three-layer urothelium and its regeneration are reduced proliferation and increased apoptosis of urothelial cells, which is accompanied by* de novo* differentiation of umbrella cells [25, 80, 89], which restore efficient blood-urine barrier [69, 79].

## 6. Conclusion

Our understanding of basic urothelial cell biology is essential for comprehension not only of normal urinary bladder functioning but also, and more importantly, of mechanisms underlying different urinary bladder disorders, including cystitis. Unique differentiation of urothelial cells with expression of specific proteins' uroplakins and their organisation into urothelial plaques ensures proper functioning of the urinary bladder as urine-blood permeability barrier in healthy individuals. In cystitis, the barrier is disrupted leading to different symptoms. Treatment of cystitis is usually restricted to symptom management, but unfortunately it is often ineffective or insufficient. New experimental tools and promising therapeutic targets represent challenging options for future research. In this respect, cyclophosphamide induced cystitis has been proven as an excellent research model. Currently, fundamental research of urothelial biology, cystitis origin, and development as well as its prevention and treatment is a rapidly expanding research field with exciting possibilities and, hopefully, considerable progress in clinical applications will soon be achieved.

## Figures and Tables

**Table 1:** Classification and clinical features of cystitis.

Category	Clinical features
Cystitis	Dysuria, urgency, frequency, lower abdominal pain, and haematuria
Uncomplicated cystitis	Cystitis in women with a structurally and functionally normal bladder
Isolated or sporadic	No cystitis symptoms in 4 weeks before this episode
Reinfection	At least 3 episodes of uncomplicated infection in past 12 months or at least 2 episodes of uncomplicated infection in past 6 months
Complicated cystitis	Cystitis in men or compromised host; cystitis with a structurally and functionally abnormal bladder
